# Dosimetric study of 2D ion chamber array matrix for the modern radiotherapy treatment verification

**DOI:** 10.1120/jacmp.v11i2.3076

**Published:** 2010-04-19

**Authors:** Sathiyan Saminathan, Ravikumar Manickan, Varatharaj Chandraraj, Sanjay. S. Supe

**Affiliations:** ^1^ Department of Radiation Physics Kidwai Memorial Institute of Oncology Bangalore India

**Keywords:** ion chamber array, intensity‐modulated radiotherapy, fluence, film dosimetry

## Abstract

Intensity‐modulated radiotherapy treatment demands stringent quality assurance and accurate dose determination for delivery of highly conformal dose to the patients. Generally 3D dose distributions obtained from a treatment planning system have to be verified by dosimetric methods. Mainly, a comparison of two‐dimensional calculated and measured data in several coplanar planes is performed. In principle, there are many possibilities to measure two‐dimensional dose distributions such as films, flat‐panel electronic portal imaging devices (EPID), ion chambers and ionization chamber arrays, and radiographic and radiochromic films. The flat‐panel EPIDs show a good resolution and offer a possibility for real‐time measurements: however to convert the signal into dose, a separate commercial algorithm is required. The 2D ion chamber array system offers the real‐time measurements. In this study, dosimetric characteristics of 2D ion chamber array matrix were analyzed for verification of radiotherapy treatments. The dose linearity and dose rate effect of the I'matriXX device was studied using 6 MV, 18 MV photons and 12 MeV electrons. The output factor was estimated using I'matriXX device and compared with ion chamber measurements. The ion chamber array system was found to be linear in the dose range of 2–500 cGy and the response of the detector was found to be independent of dose rate between 100 MU/min to 600 MU/min. The estimated relative output factor with I'matriXX was found to match very well with the ion chamber measurements. To check the final dose delivered during IMRT planning, dose distribution patterns such as field‐in‐field, pyramidal, and chair tests were generated with the treatment planning system (TPS) and the same was executed in the accelerator and measured with the I'matriXX device. The dose distribution pattern measured by the matrix device for field‐in‐field, pyramidal, and chair test were found to be in good agreement with the calculated dose distribution by TPS both for 6 and 18 MV photons (γ ≤ 1: 96%, criteria 3%, 3 mm). Two 7‐field IMRT plans (one prostate, one head and neck) dose distribution patterns were also measured with I'matriXX device and compared with film dosimetry. The measurements and evaluation proves that I'matriXX can be used for quantifying absolute dose. Moreover, using I'matriXX as absolute dosimeter in IMRT field verification, avoids the time‐consuming procedure of making ionometric measurement for absolute dose estimation and film for dose distribution verification. The I'matriXX can also used for routine quality assurance checks like flatness, symmetry, field width, and penumbra of the linear accelerator beam.

PACS number: 87.55.ne and 87.56.Fc

## I. INTRODUCTION

Intensity‐modulated fields have the potential to deliver optimum dose distributions which results in greater dose uniformity in the target and lower doses to the neighboring critical organs and normal healthy structures, as compared to conventional external beams employing wedges and cerroband blocks. Multileaf collimator (MLC)‐based intensity‐modulated radiation therapy (IMRT) can be delivered by two main modalities, namely segmental IMRT (step and shoot) and dynamic IMRT (sliding window). In the step‐and‐shoot modality, the MLC shape remains constant while the beam is on and changes while the beam is off in the sliding window, each leaf pair moves continuously, unidirectionally, and with independent speed while the beam is on. Any shape of intensity profile can be obtained by controlling the leaf movement, subject to the mechanical constraints such as leaf width, maximum speed and field size etc. imposed by the multileaf collimator (MLC) system. As leaf motions are controlled by a computer, the IMRT technique lends itself to automated treatment delivery, eliminating the need for re‐entry into the room between fields. During treatment, the leaf positions are verified by computer, ensuring better quality control than when using customized field shaping blocks.^(1)^ The implementation of IMRT in external beam therapy imposes high demands on measurement device and quality assurance. Generally three‐dimensional dose distributions obtained from a treatment planning system have to be verified by dosimetric means. Mainly a comparison of two‐dimensional calculated and measured data in several coplanar planes is performed.^(2)^ The *in vivo* determination of dose distribution inside the patient is complicated and error‐prone, while the pretreatment verification in phantom seems to be easier and more reliable. The option of the treatment planning systems that permits exporting the true radiation fields (specific for single patient) on a quality assurance phantom provides verification of the treatment delivery on a field‐by‐field basis.^(3)^

There are many possibilities to measure two‐dimensional dose distributions. The film dosimetry is a well‐established method to verify dose distributions in phantom.^(4)^ ^,^ ^(5)^ Film dosimetry permits high spatial resolution, limited only by the reading system, so that it is commonly used for measuring beam profiles and isodose curves. On the other hand, it shows a rather small dynamic range together with a nonlinear and energy dependent dose response. Moreover, film dosimetry is a very time consuming procedure if an acceptable level of accuracy is required in absolute dose determination. Radiographic and radiochromic films cannot be applied for fast real‐time measurements because their calibration and scanning processes are time‐consuming.^(2)^ Flat‐panel electronic portal imaging device (EPID) was studied by Warkentin et al.^(6)^ It has shown good resolution and offers a possibility for real‐time measurements. Nicolini et al.^(7)^ ^,^ ^(8)^ have described the GLAaS algorithm for pretreatment IMRT absolute dose verification based on the use of amorphous silicon detectors. They have concluded that the PV‐as500 detector with GLAaS algorithm is a reliable method of estimating the absolute dose. The ionization chamber array^(9)^ is capable of providing real‐time measurement and it can be easily connected to a PC with a standard Ethernet cable. The dose can be measured directly, after calibration of the ionization chamber array. Though their resolution is less, compared to that of films or EPIDs, good dosimetric agreement was noticed between films and 2‐D ionization chambers for verification of radiotherapy plans, as reported by Spezi et al. and Stasi et al.^(10)^ ^,^ ^(11)^ Due to its properties, it qualifies for measuring dynamic process, e.g. dynamic wedges, dynamic IMRT.

This study was carried out to evaluate the dosimetric performance of 2D ionization chamber array for IMRT dose verification. To quantify the performance of the device, some of the basic dosimetry tests were carried out and also some of the tests were compared with the ionization chamber measurements in phantom. The basic tests included output factor, dose linearity, and dose rate behavior. The measurements carried out by the I'matriXX device for verification of IMRT plans are also presented, and the same was compared with the film dosimetry measurements.

## II. MATERIALS AND METHODS

The I'matriXX device consists of a 1020 vented ion chamber detectors, arranged in a 32×32 grid. When irradiated, the air in the chambers is ionized. The released charge is separated by means of an electrical field between the bottom and the top electrodes. The current, which is proportional to the dose rate, is measured and digitized by a non‐multiplexed 1020 channels current sensitive analog to digital converter. The each chamber volume is $0.08{\text{cm}}^{3}$ with the height of 5 mm and diameter of 4.5 mm. The spatial resolution of the detector system is 7.5 mm. The OmniPro IMRT software can able to give 1 mm resolution with linear interpolation using low pass filter. The maximum dose rate detectable by the detectors are 5 Gy/min and minimum detectable dose rate is 0.1 Gy/min.^(12)^ The bias voltage required for the I'matriXX system is 500±30V. The equivalent absorber thickness on the front side of the matrix is 3.6 mm. The maximum field of view is $24\times 24\;{\text{cm}}^{2}$. Before taking the measurement, the device requires a 15 min warm‐up time. The detector panels are cooled by forced air cooling via two fans. The device runs with two separate counters to avoid dead time, the minimum sampling period is 20 mins. The matrix device can be directly connected to the PC via standard ethernet interface to acquire the measurement.

**Figure 1:**
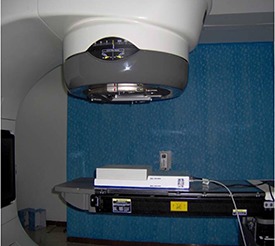
Measurement setup at the accelerator with I'matriXX.

The measurements were performed in Clinac DHX linear accelerator with 6 and 18 MV photons and 12 MeV electrons. The I'matriXX was irradiated with $10\times 10\;{\text{cm}}^{2}$ field size and source to effective point of measurement (3.6 mm from surface) distance was maintained as 100 cm with 5 cm solid water (RW3) phantom as a buildup material and 5 cm solid water as a backscatter material (Fig. 1).

### A. Dose and dose rate dependence

The dose linearity test was performed using 6 and 18 MV X‐ray photons by irradiating I'matriXX device with $10\times 10\;{\text{cm}}^{2}$ field size. The measurements were carried out at 100 cm FSD and the detector plane was maintained at 5 cm depth. The detector linearity was verified by measuring the I'matriXX output for 2, 3, 5, 10, 20, 50, 100, 200, 300 and 500 MUs set on the accelerator console. The dose rate effect dependency of I'matriXX was tested for both 6 and 18 MV photons for different dose rates ranging from 100 MU/min to 600 MU/min for the same set monitor unit on the console. The dose and dose rate effect of I'matriXX was also tested with 12 MeV electrons at reference depth ($2.89g/{\text{cm}}^{2}$) using an applicator of $15\times 15\;{\text{cm}}^{2}$.

### B. Output factor

The normalized output factor was estimated using I'matriXX device by maintaining 100 cm FSD and positioning the detector plane at 5 cm depth. Efficiently this test assessed the scatter properties of the I'matriXX detectors, which depend on the internal design of the device. The response of 2D ion chamber array for a small radiation beams was of particular interest because of its potential applications to the verification of IMRT plans. The output factor measurements were carried out by delivering 100 MU for square field sizes ranging from $3\times 3\;{\text{cm}}^{2}$ to $24\times 24\;{\text{cm}}^{2}$. The I'matriXX signal was averaged over the 2×2 central pixels which cover the area of the central four reference ionization chambers. Dose outputs were compared with ion chamber measurements carried out with the same geometry using RW3 solid water phantom.

### C. Comparison between dose distributions calculated by Eclipse planning system and measured with the I'matriXX and film dosimetry system

In order to verify an IMRT plan, a verification plan was produced for every original plan in the treatment planning system. The CT data of the measurement system was used to estimate the dose distribution at depth for these verification plans. The I'matriXX device with 5 cm solid water phantom positioned above and below was scanned with 2 mm CT slice thickness. The verification plan was exported to the scanned detector system with the detector plane positioned at isocentre. In the verification plan, all the fields used for the planning where set at 0 degree gantry and collimator angles. The central beam was made perpendicular to the I'matriXX measurement level at the center of the measurement area. Within the treatment field, the dose at the detector plane was calculated and transferred to OmniPro IMRT software for comparison. Every verification field was exported to the accelerator console, and the same was delivered and measured by the I'matriXX device. All individual field delivered dose distribution patterns were added to obtain the composite dose distribution, and the same was compared with the TPS generated composite dose distribution using the Gamma Index Method. The spatial difference in the dose distribution (Distance‐To‐Agreement) and the dose deviation (Delta‐Dose) are verified with the Gamma Index Method at a particular point simultaneously. The generated resulting matrix gives the value of Gamma Index for every tested measurement points. If this value is smaller than or equal to 1, the criteria values are not exceeded. When the Gamma Index value is higher than 1, the measurement result lies outside the tolerance range.

The measurement was also carried out in the solid water phantom (RW3) using EDR2 verification film. The film was positioned at 5 cm depth in solid water phantom with 10 cm of scattering material present at the bottom. The above phantom set was CT scanned similar to I'matriXX phantom to create a verification plan in the Eclipse treatment planning system.

Similarly, the test patterns generated by treatment planning system, like chair test and pyramidal test, were exported to verification phantoms for quantitative evaluation with I'matriXX and film dosimetry systems. Two 7‐field IMRT plans (one prostate, one head and neck) were also considered for comparing the calculated and measured dose distributions.

## III. RESULTS & DISCUSSION

### A. Dose and dose rate dependence

**Figure 2:**
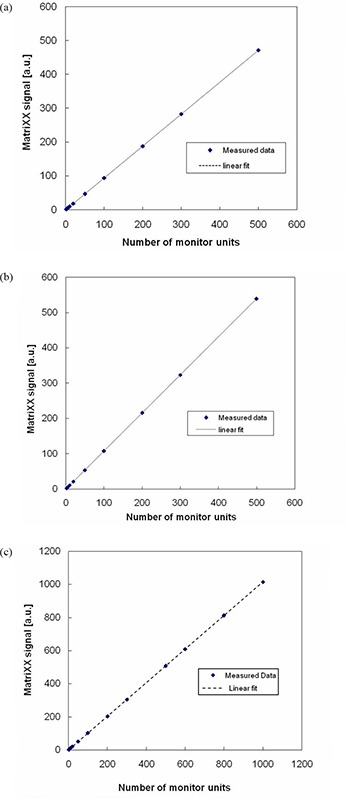
The signal of the I'matriXX detector for different number of monitor units for: a) 6 MV photons; b) 18 MV photons; c) 12 MeV electrons.

**Figure 3:**
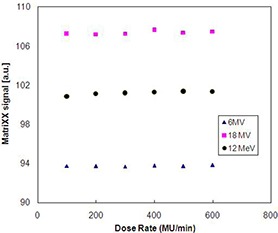
The signal of I'matriXX detector for different dose rates for 6 and 18 MV photons and 12 MeV electrons.

The response of the I'matriXX device (arbitrary units) was found to be linear with dose in the clinically useful range of 2 to 500 MU for both 6 and 18 MV photons and 12 MeV electrons (Fig. 2 (a), (b), (c)). Also, the I'matriXX array detectors were found to be independent of dose rate between 100 MU/min to 600 MU/min for both 6 and 18 MV photons and 12 MeV electrons (Fig. 3). The observed results were in close agreement with ion chamber measurements. As the response of the I'matriXX device is linear with dose and dose rate, it can be used for the measurement of dose gradients effectively.

### B. Output factor

**Figure 4:**
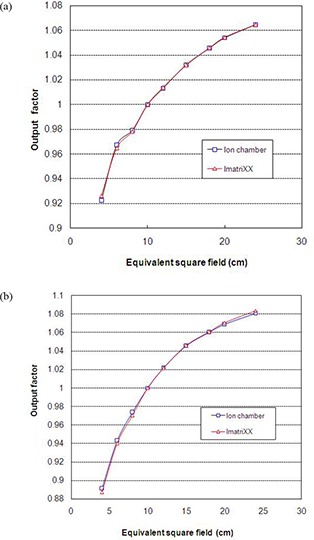
Output comparison between I'matriXX (Δ) and ion chamber measurements (□) for: a) 6 MV photons; b) 18 MV photons.

The output factors estimated using the array detectors were found to be in close agreement with the output factors measured using $0.6{\text{cm}}^{3}$ ionization chamber (Fig. 4 (a), (b)) for 6 MV and 18 MV photons. The measured output factor for 12 MeV electrons were also in close agreement with the parallel plate ionization chamber measurement. Small discrepancies of the order of ±0.5% noticed could be due to the scatter arising from the components of I'matriXX.

### C. Comparison between dose distribution calculated by Eclipse planning system and measured with the I'matriXX and film dosimetry system

Figures 5(a)) and (b) show comparison of TPS calculated and measured chair test pattern by I'matriXX and film dosimetry system. Comparison of TPS generated dose distribution with the dose distribution measured using film has resulted in the pixel match of 97.13% with γ≤1, and a pixel match of 97.86% with γ≤1 was noticed for TPS and I'matriXX dose distribution pattern (for 3% delta dose and 3 mm DTA). Figures 6(a)) and (b) show comparison of TPS calculated and measured pyramid test pattern by I'matriXX and film dosimetry system. The pixel match of 96.67% for γ≤1 was observed for dose distribution generated using TPS generated and measured with film. The pixel match of 96.36% with γ≤1 was noticed between TPS generated and I'matriXX measured dose distribution pattern (3% delta dose and 3 mm DTA).

**Figure 5(a):**
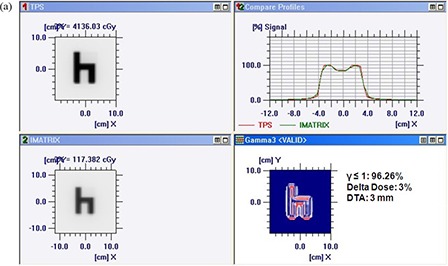
Comparison of TPS‐generated and I'matriXX‐measured chair test pattern.

**Figure 5(b):**
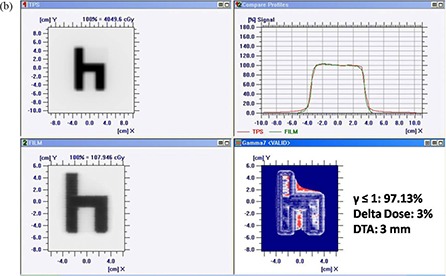
Comparison of TPS‐generated and film‐measured chair test pattern.

Figures 7(a) and (b) show the comparison of measured and calculated dose distribution pattern for seven‐field prostate patient. The match result has shown 97.43% agreement for γ≤1 with 3% delta dose and 3 mm DTA for TPS generated and I'matriXX measured dose distribution patterns, and 98.02% for TPS generated and film measured dose distribution patterns. Figures 8(a) and (b) show the comparison of measured and calculated dose distribution pattern for seven‐field head and neck patient. The match result has shown 97.3% agreement for γ≤1 with 3% delta dose and 3 mm DTA for TPS generated and I'matriXX measured dose distribution patterns, and 95.13% for TPS generated and film measured dose distribution patterns. The comparison of gamma pixel match for I'matriXX and film with the TPS calculated dose distribution for various test patterns are summarized in Table 1. The mismatch of high gamma value regions (>1) could be due to the large dose gradient in the delivered dose and the limited resolution of the measuring system to detect the same.

**Table 1:** Summary of the results of gamma pixel match.

	*Gamma Pixel Match (* γ≤1 *) for 3% Delta Dose & 3 mm DTA*
Chair Test (TPS vs. I'matriXX)	97.86%
Chair Test (TPS vs. Film)	97.13%
Pyramidal Test (TPS vsx I'matriXX)	96.36%
Pyramidal Test (TPS vs. Film)	96.67%
Prostate (TPS vs. I'matriXX)	97.43%
Prostate (TPS vs. Film)	98.02%
Head & Neck (TPS vs. I'matriXX)	97.30%
Head & Neck (TPS vs. Film)	95.13%

**Figure 6(a):**
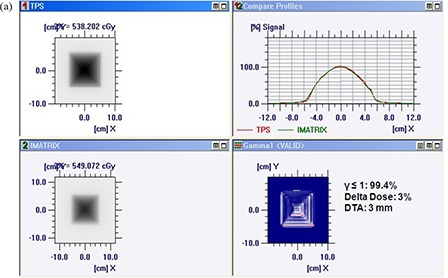
Comparison of TPS‐generated and I'matriXX‐measured pyramid test pattern.

**Figure 6(b):**
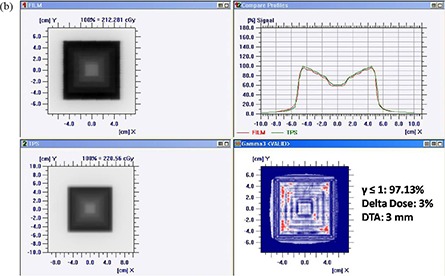
Comparison of TPS‐generated and film‐measured pyramid test pattern.

**Figure 7(a):**
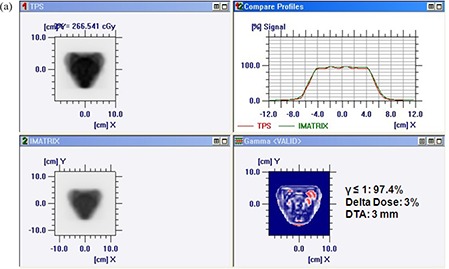
Comparison of TPS‐generated and I'matriXX‐measured dose distribution for seven‐field prostate plan.

**Figure 7(b):**
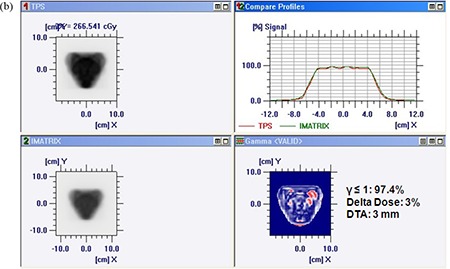
Comparison of TPS‐generated and film‐measured dose distribution for seven‐field prostate plan.

**Figure 8(a):**
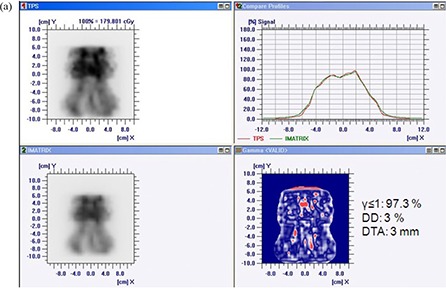
Comparison of TPS‐generated and I'matriXX‐measured dose distribution for seven‐field head and neck plan.

**Figure 8(b):**
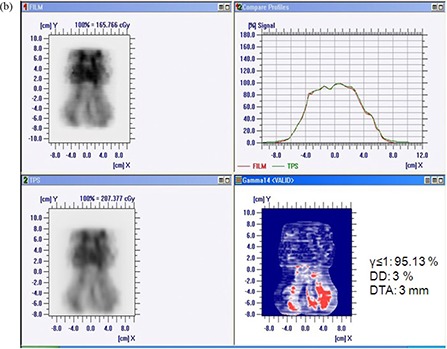
Comparison of TPS‐generated and film‐measured dose distribution for seven‐field head and neck plan.

## IV. CONCLUSIONS

IMRT has become one of the commonly used radical treatment technique. Presently, unless the absolute dose is measured and checked individually, the treatment outcome cannot be assessed. The 2‐D ion chamber array matrix is one of the easy to use QA tool for IMRT technique. To quantify the performance of the device, some of the basic dosimetry tests were carried out and also some of the tests were compared with the ionization chamber measurements in phantom. The basic test includes dose linearity, dose rate behavior, and output factor. The performance of I'matriXX device in verification of IMRT plans are also presented and compared with the film dosimetry system. The measurements and evaluation processes with I'matriXX has shown that it can be used for quantifying absolute dose with the required accuracy level. As the accuracy of dose measurement with I'matriXX is good, the need for depending upon time‐consuming measurements with film or ion chamber can be avoided. The detecting system was found to be linear with dose and independent of dose rate. Our measurement results were comparable with the results reported in the literature.^(2)^

The measured and TPS calculated dose distribution patterns were in good agreement for test patterns and the patient treatment plans. Though the film dosimetry system gives good resolution, it is labor‐intensive procedure, whereas the I'matriXX can be used as a direct reading device. The measurement with I'matriXX takes less time compared to film dosimetry system for IMRT field verification. The I'matriXX can also be used for routine quality assurance checks such as flatness, symmetry, field width, and penumbra checks of the linear accelerator beam.
